# Automated Stock Volume Estimation Using UAV-RGB Imagery

**DOI:** 10.3390/s24237559

**Published:** 2024-11-27

**Authors:** Anurupa Goswami, Unmesh Khati, Ishan Goyal, Anam Sabir, Sakshi Jain

**Affiliations:** Remote Sensing Lab, Department of Astronomy, Astrophysics and Space Engineering, Indian Institute of Technology, Indore 453552, India; unmesh.khati@iiti.ac.in (U.K.); goyalishan@iiti.ac.in (I.G.); phd2201121003@iiti.ac.in (A.S.); phd2201121014@iiti.ac.in (S.J.)

**Keywords:** UAV, object segmentation, deep learning, Detectree, tree crown area, stock volume, above-ground biomass (AGB)

## Abstract

Forests play a critical role in the global carbon cycle, with carbon storage being an important carbon pool in the terrestrial ecosystem with tree crown size serving as a versatile ecological indicator influencing factors such as tree growth, wind resistance, shading, and carbon sequestration. They help with habitat function, herbicide application, temperature regulation, etc. Understanding the relationship between tree crown area and stock volume is crucial, as it provides a key metric for assessing the impact of land-use changes on ecological processes. Traditional ground-based stock volume estimation using DBH (Diameter at Breast Height) is labor-intensive and often impractical. However, high-resolution UAV (unmanned aerial vehicle) imagery has revolutionized remote sensing and computer-based tree analysis, making forest studies more efficient and interpretable. Previous studies have established correlations between DBH, stock volume and above-ground biomass, as well as between tree crown area and DBH. This research aims to explore the correlation between tree crown area and stock volume and automate stock volume and above-ground biomass estimation by developing an empirical model using UAV-RGB data, making forest assessments more convenient and time-efficient. The study site included a significant number of training and testing sites to ensure the performance level of the developed model. The findings underscore a significant association, demonstrating the potential of integrating drone technology with traditional forestry techniques for efficient stock volume estimation. The results highlight a strong exponential correlation between crown area and stem stock volume, with a coefficient of determination of 0.67 and mean squared error (MSE) of 0.0015. The developed model, when applied to estimate cumulative stock volume using drone imagery, demonstrated a strong correlation with an R^2^ of 0.75. These results emphasize the effectiveness of combining drone technology with traditional forestry methods to achieve more precise and efficient stock volume estimation and, hence, automate the process.

## 1. Introduction

Forest ecosystems are the largest aboveground carbon reservoir in the terrestrial biosphere and play a critical role in regulating global carbon dynamics [1]. Through processes like degradation and regrowth, they significantly impact carbon storage, which is essential for maintaining climate balance and biodiversity [2]. The parameters highlighted in this study are stock volume and above-ground biomass, which are key indicators in forest inventories that play a crucial role in effective forest functions and management [3]. The traditional and indirect ground-based method for calculating them is DBH, which is often labor-intensive, time-consuming, and limited in scope, making them less effective for large-scale forest assessments [4,5].

So, to solve this problem, advancements in forestry research have enabled the usage of UAVs. This is beneficial for measuring forest characteristics over a specific area, providing the ability to operate in a wide range of environments, the flexibility to conduct periodic surveys, and the capability to fly at low altitudes capturing extremely high-resolution images [6]. They have become invaluable tools due to their cost-effectiveness, enabling the assessment of spatially explicit inventories across various scales, including hard-to-reach areas [7,8]. Ultimately, with these benefits, precision forestry has evolved for effective forest studies [9].

As mentioned earlier, making tree diameter measurements on the ground can be tough; hence, drones are being used for this purpose, which is essential for stock volume and biomass computations. To calculate DBH, ultra-fine resolution orthophotos, stereo pair images, and 3D models are generated using Structure from Motion and aerial photogrammetry [10]. As another research aspect, drones are used to study some of the tree characteristics that can be linked with trunk diameter to build an understanding of the forest parameters. One of its important parameters is detecting and delineating the tree crowns. Four renowned algorithms are used for this process: (i) Individual Tree Crown Detection (ITCD) [11]; (ii) TIDA (Tree Identification and Delineation Algorithm), which employs a ‘top-down’ spatial clustering approach to automate tree crown delineation by analyzing variations in crown reflectance profiles [12]; (iii) the tree crown segmentation algorithm, which utilizes multispectral imagery by generating a morphological gradient map [13]; and (iv) the delineation method, which is used in this work at three spatial scales: superpixels, conventional multiresolution segmentation objects, and DeepForest-detected bounding boxes [14,15]. Two of the accuracy assessment algorithms are developed to quantify the accuracy of the delineation technique. One is a synthetic test that helps to develop statistical metrics [16]. The second is MCG-Tree, a graph-based approach that addresses over-segmentation by combining geometric and spectral criteria [17].

Post crown detection and delineation, parameters like tree canopy area and diameter are computed. This is carried out using canopy height models, digital surface models, and object-based image analysis [18,19]. After estimating the canopy cover area and the DBH, stock volume estimation is performed using deep learning algorithms. One such algorithm is BlendMask, which works on the Mask R-CNN architecture [20,21]. It is useful for object segmentation [22]. Another technique utilized is a pre-trained UNET network for semantic segmentation [23]. Machine learning models, such as auto-regressive moving averages and long short-term memory, are also used to estimate the stock volume [24]. It is also computed using methods such as terrestrial laser scanning and airborne LiDAR, where tree height and DBH are calculated through regression analysis [25,26].

Previous works have mapped the relationship between DBH and stock volume, providing valuable insights for understanding forest parameters. Also, in a prior research work, stock volume was also computed directly without field measurements, relying on airborne lidar. This was carried out using traditional allometric equations, the known DBH, species identification, and height data [27]. However, not much work has been conducted to measure tree crown area and directly estimate stock volume and above-ground biomass using drone RGB imagery.

This work aims to draw a relationship between the DBH, stock volume, and tree crown area and develop an empirical model to compute stock volume using the canopy cover area. In this work, tree crown area estimation is carried out using machine learning algorithms like Detectree2 and DeepForest, which operate on the Mask R-CNN computer vision framework. To contribute to the advancement of this field, this research uses UAV RGB imagery to focus on the following:

- Performing tree crown segmentation for crown area estimation;
- Finding the empirical relationship between the tree crown area and stem stock volume, contributing to a better understanding of forest ecology;
- Outlining a methodology for automatically predicting stock volume based on tree crown segmentation using UAV data.

Moreover, understanding the empirical relationship between the tree crown area and stem stock volume will further help this study to estimate above-ground biomass, which is crucial for carbon stock assessments, hence offering new insights that could enhance sustainable forest management practices.

## 2. Methods

**Figure 1:**
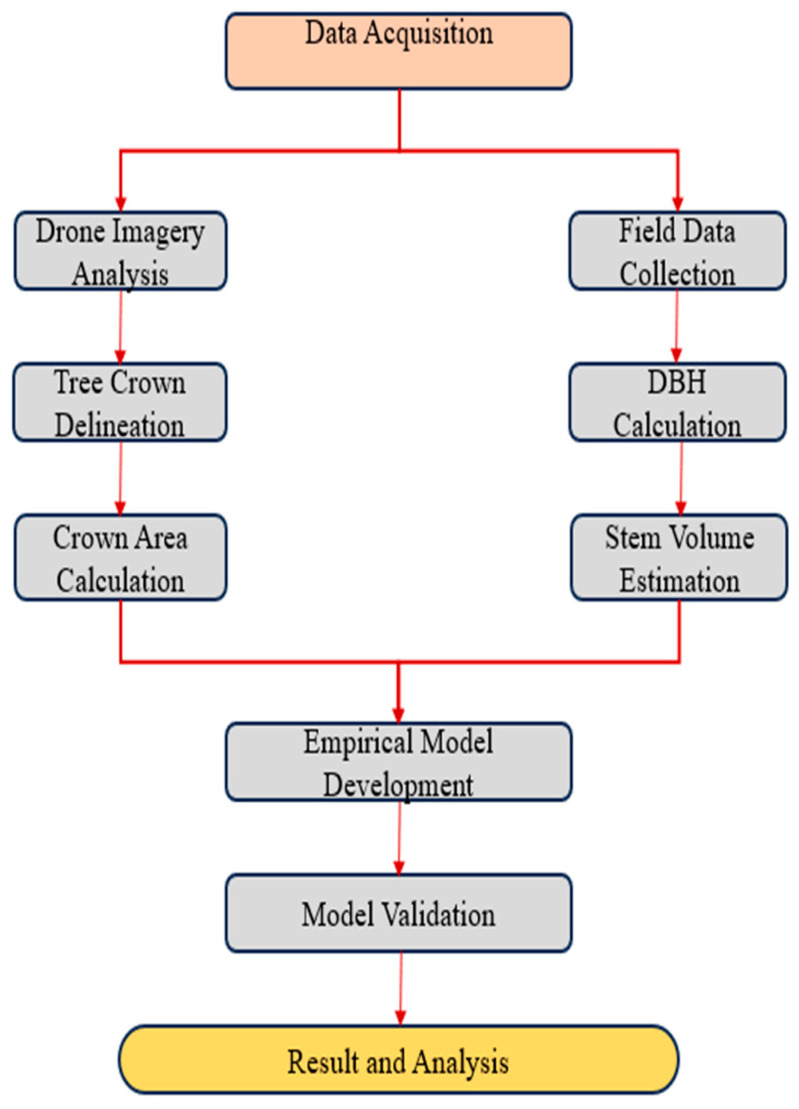
A flowchart of the methodology for automated stock volume estimation.

This study followed a phased approach, starting with tree crown delineation and area estimation using drone imagery, followed by stock volume estimation through allometric equations and empirical modeling. An overview of the methodology is shown in Figure 1. Tree canopies were extracted from UAV RGB data in two steps: image segmentation based on object bounding box and class and box offset using Detectron2. Ground data and extracted features were used for stock volume and AGB estimations, along with validation.

### 2.1. Study Site

**Figure 2:**
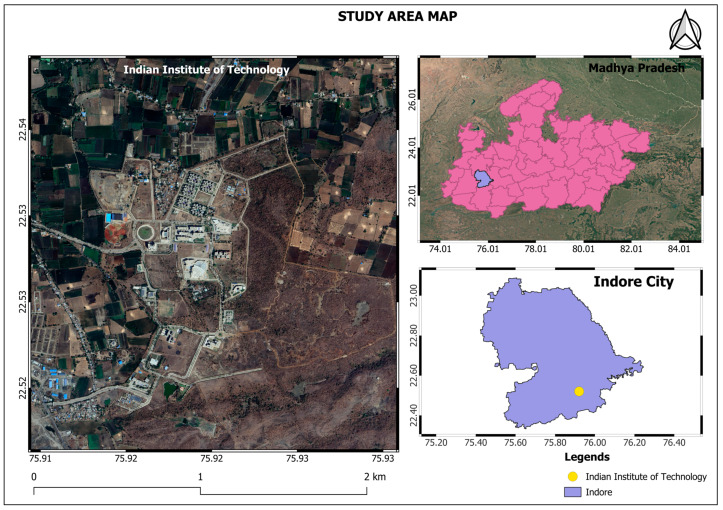
Study area location map. This map shows the study area of the Indian Institute of Technology, which is situated in Indore city in the state of Madhya Pradesh.

This study was conducted at the Indian Institute of Technology Indore (Figure 2), Madhya Pradesh, India, located at 22°31′21″ N and 75°55′48″ E, with an elevation of approximately 600 m above sea level. It falls in the western part of Malwa plateau. The study site experiences a tropical wet and dry climate, with distinct seasonal variations. Temperatures range from a low of 2 °C to a high of 41 °C. Annual rainfall is approximately 1600 mm, primarily occurring during the southwest monsoon. IIT Indore has 198 acres of forest land that features a range of tree species, including Teak (*Tectona grandis*), Neem *(Azadirachta indica*), Mango (*Mangifera indica*), and Banyan (*Ficus benghalensis*), providing an ideal environment for aerial remote sensing to assess tree cover. The vegetation in the study area included dense Teak wood (Tectona grandis), which is well known as a woody material with various uses due to its high quality and natural durability [28].

### 2.2. Data Acquisition and Processing

**Figure 3:**
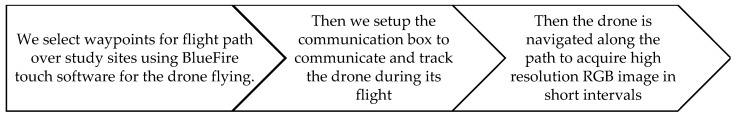
Drone data acquisition flowchart.

**Figure 4:**
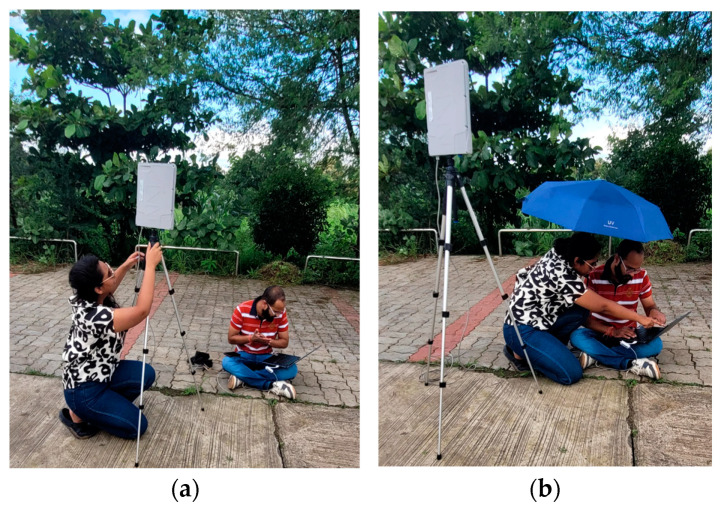
Data collection using the drone. The figure (**a**) shows the setup of the communication box for the real-time tracking of the drone. Figure (**b**) shows the flight planning using BlueFire Touch software of v4.1.9047.1979 for the drone. During this stage, the waypoints for the drone flight were decided.

The datasets used in this study included RGB images taken with a Sony ILCE-5100 camera that were captured by a flying UAV Ryno-Lite Surveying Mapper. The data acquisition process using drones is outlined in Figure 3. The UAV was flown at an altitude of 100 m above ground level, providing an optimal balance between spatial resolution and coverage area. The sensor had a 16 mm focal length, with compact dimensions of 62 × 22.5 mm, and a lightweight design of 67 g. The drone was equipped with a communication box that enabled real-time communication (Figure 4) and precise location tracking. With an optimal operational range, the drone covered the study area in 3 flights.

**Figure 5:**
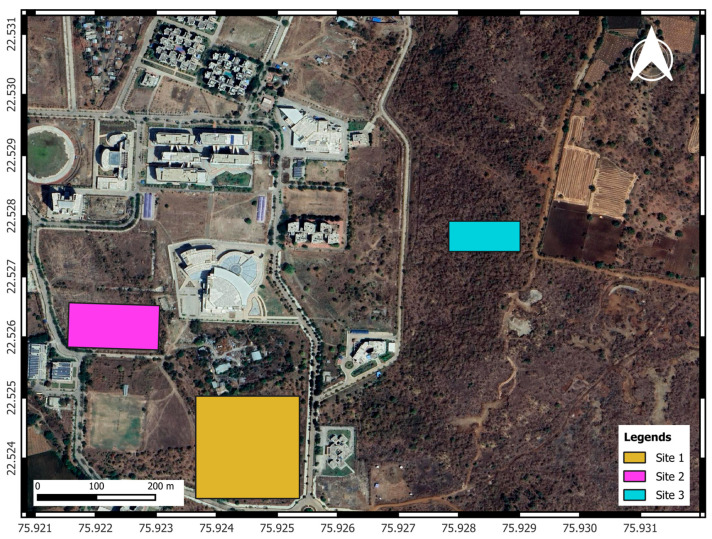
Three different study sites were identified during this study. The locations where the drone imagery of the tree-covered areas was captured are shown here.

Three sites were selected for this study (Figure 5). All these sites were dominantly covered by Teak trees.

**Figure 6:**
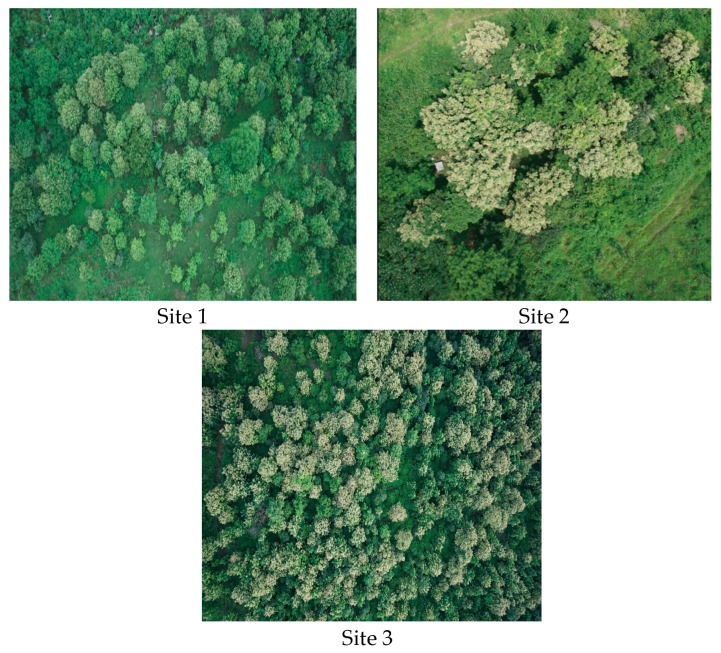
Images of tree canopies captured by the drone at sites 1, 2, and 3.

Each image captured (Figure 6) had a resolution of 6000 × 4000 pixels with a ground sampling distance for each pixel of 0.025 × 0.025 m, ensuring the high detail essential for precise analysis.

**Figure 7:**
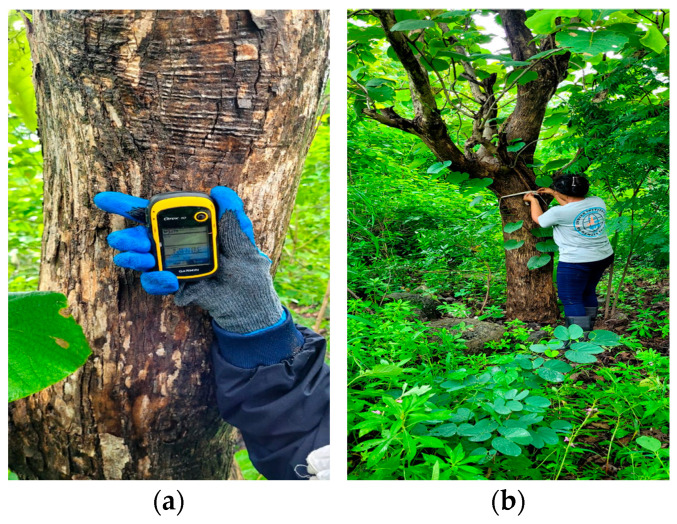
Field work performed for collecting DBH values. Figure (**a**) shows the geographic location data collection carried out using GARMIN eTrex 10, and Figure (**b**) depicts the DBH measurement of the tree trunks.

Fieldwork involved measuring the circumference of each tree trunk at breast height using a measuring tape and recording its geographic coordinates with a GPS device (Figure 7). A total of 125 trees were identified and marked on the ground, which were captured by the drone at site 1 and site 2. Each tree trunk was measured to find out the DBH. For the third site, there were 7 plots, each measuring 25 × 25 m. A total of 450 trees were measured manually for the ground truth values.

### 2.3. Tree Crown Delineation Using Detectron2 and Crown Area Computation

**Figure 8:**
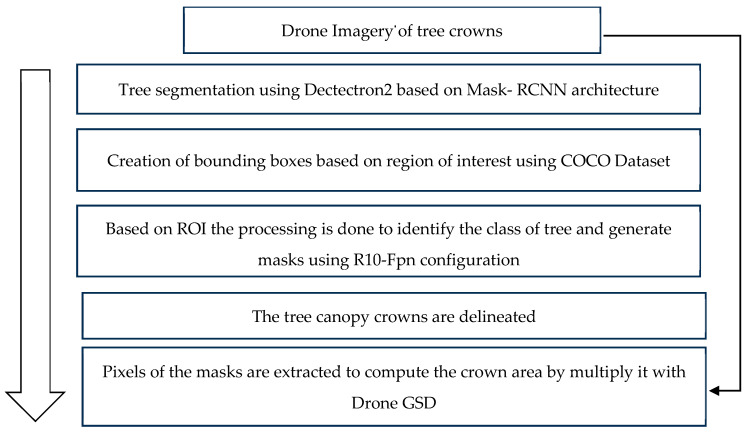
Flowchart for tree crown delineation methodology.

The tree canopies were segmented using the mentioned method based on the drone imagery (Figure 8). Object detection was combined with object segmentation through image segmentation. After an object was detected, a bounding box was generated around the object. Segmentation was carried out to identify those pixels that corresponded to the object of interest. Here, the Detectron2 deep learning model was used, which provided deep learning detection and segmentation algorithms. It is based on Facebook AI’s Mask RCNNN architecture, which is based on the Faster R-CNN architecture, which performs object detection using a Region Proposal Network (RPN). An RPN is a fully convolutional network that is trained end-to-end to generate Regions of Interest (ROIs) for each image. These RoIs were accompanied by object bounds indicating the boundaries of the RoI and the likelihood that it contained an object. The RoIs were then processed through fully connected convolutional networks to identify the class of the object within each bounding box and to produce an exact mask for each object [29]. The Detectron2 computer vision library was used to handle geospatial data and perform the delineation of individual tree crowns. This library executes instance segmentation by generating object ‘masks’ that precisely outline the objects within an image. From “model zoo”, a specific pre-trained model architecture with weights was used, “DetectreeRGB”, in which the R10-FPN configuration was used. This architecture combines a 101-layer-deep ResNet module with a Feature Pyramid Network module. The configuration set the backbone of the network, which is the part that views and extracts features from the scene. Each segmented feature has a confidence score attached to it that relates to how much the network is sure about the object of interest. A suitable threshold was selected to optimize accuracy. The pre-trained dataset used for this work was COCOs (Common Objects in Context), which is a large-scale image recognition dataset used for segmentation, providing 330,000 images as training data [30]. Once individual tree crowns were delineated, their areas were calculated by extracting the number of pixels covered by each crown and multiplying this count by the ground sampling distance of the drone camera sensor, which was 0.025 m.

### 2.4. Stock Volume Estimation

**Figure 9:**
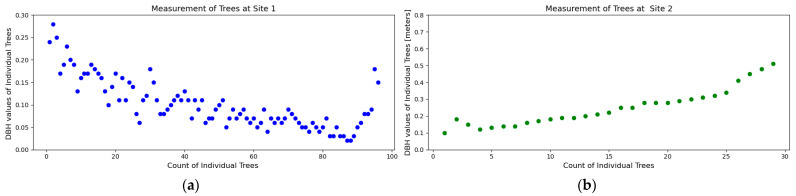
(**a**) and (**b**) show the measurement of DBH computed from the filed observations. (**a**) shows site 1, and (**b**) shows site 2.

The second phase focused on stock volume estimation and involved extensive field data collection. A total of 125 Teak trees, which were detected using the drone imagery, were selected across two sites. The total volume was predicted using the DBH value. For computing the DBH, we measured the circumference of the trunk at breast height (1.37 m from the ground level). The main bark was measured after removing all the branches. The measurements were calculated in centimeters. The DBH values are shown below (Figure 9). Geographic coordinates were recorded for each tree.

Subsequently, the stem volume was computed using Equation (1) based on the calculated DBH values denoted by *D*. Equation is defined as follows:

(1) $${V=\left(-0\cdot 1163+2\cdot 8013*D\right)}^{2}$$

***Equation (1)**: Formula to calculate the stem stock volume using the DBH.*

### 2.5. Model Development for the Automated Stock Volume Computation

To establish a relationship between crown area and stem volume, an empirical model was developed. In the data, the tree crown area was the independent variable (x) and the tree stem volume was the dependent variable (y). To ensure data quality, an outlier detection method using Z scores with a threshold of 2 was applied. This filtering process helped us to remove extreme values that could potentially skew the analysis. An exponential function was chosen to model the relationship between the tree crown area and stem volume, expressed as y = a × exp (b × x), where y represents the tree stock volume and x represents the tree crown area. Here, the data for the area of the tree crowns from the drone and the stock volume calculated using the DBH from the field were used. After the stock volume was computed, the above-ground biomass was calculated by multiplying the stock volume by the wood density, which was 0.57 g per cubic meter, followed by the carbon stock, which comprised 0.43% of the total above-ground biomass, and the carbon dioxide emission was calculated by multiplying the molecular weight of carbon dioxide by the carbon stock.

For validation purposes, the dataset was then strategically split, with 70% allocated for model training and the remaining 30% reserved for testing purposes. This division ensured a proper model development process while maintaining a sufficient portion for validation. Ten random iterations were performed to check the accuracy of the model developed. The same procedure was followed for above-ground biomass estimation, with R^2^ being calculated. The model’s performance was evaluated using several metrics: the mean squared error (MSE), the mean absolute error (MAE), and the coefficient of determination (R^2^). These metrics provided a comprehensive assessment of the model’s accuracy and predictive power. The final step in the methodology was the accuracy of the automated stock volume estimation using our model being tested. This involved seven plots, each measuring 25 m × 25 m. Approximately 100 tree canopies were delineated using the machine learning algorithm, which had a confidence level between 35 and 100%. Then, the crown area was calculated, followed by stock volume calculation using our developed model.

These plots had field-measured stock volumes that were then compared with the volumes computed by our developed model, providing a comprehensive assessment of the model’s accuracy and reliability.

## 3. Results

### 3.1. Tree Crown Delineation Using Detectron2 and Crown Area Computation

**Figure 10:**
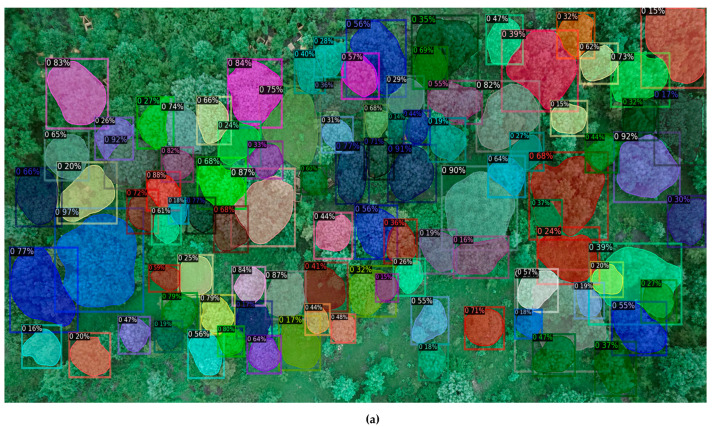

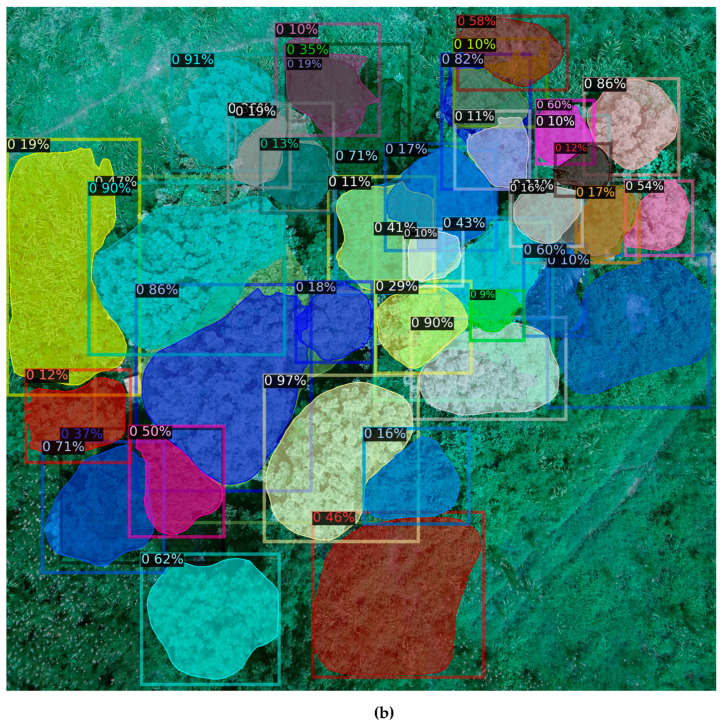
Tree crown delineation from the drone imagery for site 1 (**a**) and tree crown delineation from the drone imagery for site 2 (**b**).

On performing tree crown delineation at two different sites, 90 and 35 trees were segmented, respectively (Figure 10a,b). The segmentation occurred along the edges of each tree crown detected in the drone imagery. After tree crown delineation, the crown cover areas were computed, along with the circumferences of the respective tree trunks from the fieldwork. Here, the analysis included 125 trees, which excluded 10 trees from the actual dataset, which were identified as false positives or overlapping tree crowns.

#### Tree Crown Area Correlation with Different Tree Parameters

**Figure 11:**
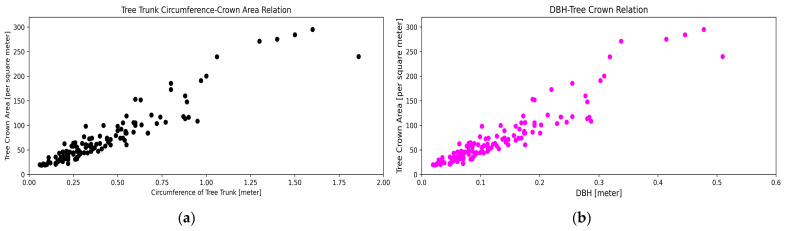
(**a**) shows the relationship between the tree trunk circumference and crown area. (**b**) shows the relationship between the DBH and tree crown.

The scatter plot (Figure 11a) represents a positive correlation between the tree crown area and the trunk circumference. For trunk circumferences below 0.25 m, crown areas remain relatively small (0–50 square meters). However, as the trunk circumference increases beyond 0.25 m, the crown area exhibits exponential growth. Between 0.25 and 1.25 m, crown areas range from 75 to 200 square meters. For trees with circumferences exceeding 1.25 m, crown areas typically fall between 250 and 300 square meters.

From these observations, now, the DBH is calculated from the trunk circumference. For the circumference in the range up to 1.75 m, the DBH ranges between 0.02 and 0.5 m. From the obtained DBH values, the stock volume of the trees (*Tectona Grandis*) is calculated. The scatter plot (Figure 11b) indicates an exponential increase in the stock volume with an increasing DBH. For DBH values below 0.2 m, the stock volume remains below 0.25 cubic meters. In contrast, the stock volume grows exponentially to 1.75 cubic meters as the DBH rises from 0.2 to 0.5 m. Now, further looking into the relationship between the DBH and tree crown area, the scatter plot reveals a strong nonlinear relationship between them. As the DBH increases, the rate of growth in the tree crown area accelerates, indicating an exponential trend. The crown area of the tress rapidly increases between 150 and 300 square meters for the DBH of 0.2 to 0.4 m. A nominal increase in the readings is observed below and above this range.

### 3.2. Model Development

**Figure 12:**
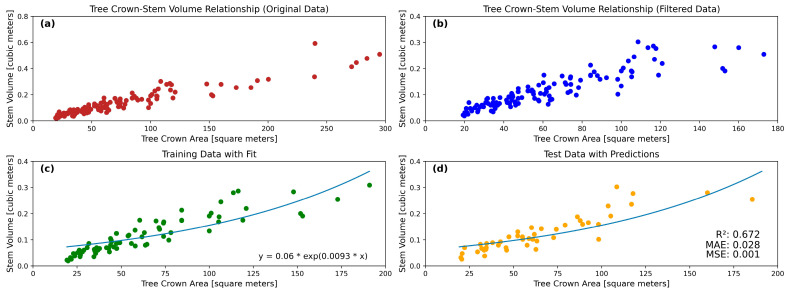
(**a**) shows the relationship between tree crown and stem volume. (**b**) shows the crown area data filtered. (**c**) shows the training data points for the model. (**d**) shows the testing data points of the model developed.

Moving forward towards achieving the main objective of this study, the scatter plot (Figure 12) shows the relationship between the tree crown area in square meters and the tree stem volume in cubic meters. The data points indicate a positive correlation between the two variables. As the tree crown area increases, the tree stem volume also tends to increase. Most of the tree crown areas range from 50 to 150 square meters, with corresponding stem volumes being generally between 0.1 and 0.3 cubic meters. Some larger crown areas up to 300 square meters have even higher stem volumes, approaching 0.6 cubic meters. This trend suggests that trees with larger crown areas tend to have higher stem volumes, reinforcing the potential for using the crown area as a predictor for stem volume in forest inventory assessments.

A statistical measurement for filtering the data was applied by computing the Z score for the datasets. After removing the outliers, the tree count number was reduced from 125 to 116 points. After the outliers’ removal, the scatter plot (Figure 12b) shows that the tree crown areas range from 25 to 175 square meters, with corresponding stock volumes between 0.05 and 0.35 cubic meters.

The dataset was then divided into training and testing sets in a 70:30 ratio to train and evaluate the model, resulting in the optimal empirical equation that best fits the data (Figure 12c). The Equation (2) hence achieved was

(2) $$y=0.06{\mathrm{e}}^{0.0093x},\;\mathrm{where}\;x\;\mathrm{is}\;\mathrm{the}\;\mathrm{tree}\;\mathrm{crown}\;\mathrm{area}$$

***Equation (2)**: Empirical equation to relate stock volume and tree crown area.*

The model’s performance was evaluated on the testing set using the RMSE (root mean squared error), which was calculated to be 0.0386, while MSE was 0.0015 and MAE was 0.0281, and the coefficient of determination was evaluated to be 0.6722 (Figure 12d).

**Figure 13:**
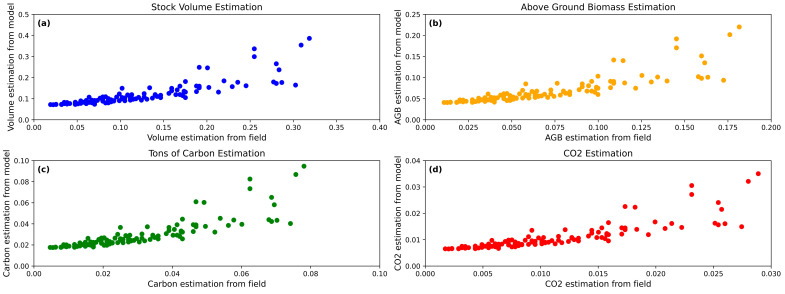
(**a**) shows the values of stock volume from field measurements on the x-axis and for the model on the y-axis. (**b**) shows the values of the AGB from field measurements on the x-axis and for the model on the y axis. (**c**) shows the values of ton carbon from field measurements on the x-axis and the model in the y-axis (**d**) shows the values of tons/ha CO_2_ emissions from field measurements on the x-axis and for the model on the y-axis.

After the stock volume was estimated using the model the AGB, carbon stock estimation and carbon dioxide emission were also calculated and then compared with the measured parameters from the field for the 125 tree samples (Figure 13). The results demonstrated that the above-mentioned parameter estimations from the field and the model were almost the same. The stock volume was calculated (Figure 13a) in the field to have a maximum value of 0.32 cubic meters, while the model’s maximum was estimated to be 0.35 cubic meters. The R^2^ and RMSE were noted to be 0.41 and 0.07, respectively. The AGB was found to be 0.175 tons/ha in the field and 0.25 tons/ha using the model (Figure 13b), with an RMSE of 0.07. For ton C/ha, Figure 13c values ranged between 0 and 0.08 for field measurements and between 0.01 and 0.1 for model calculations, with an RMSE of 0.01. For ton/ha CO_2_, the values (Figure 13d) ranged between 0.001 and 0.03 for field measurements and between 0.009 and 0.4 for model calculations, with an RMSE of 0.007. The overall R^2^ was 0.41. This shows that the model gives good estimation values for the given forest parameters.

*Validation*

**Figure 14:**
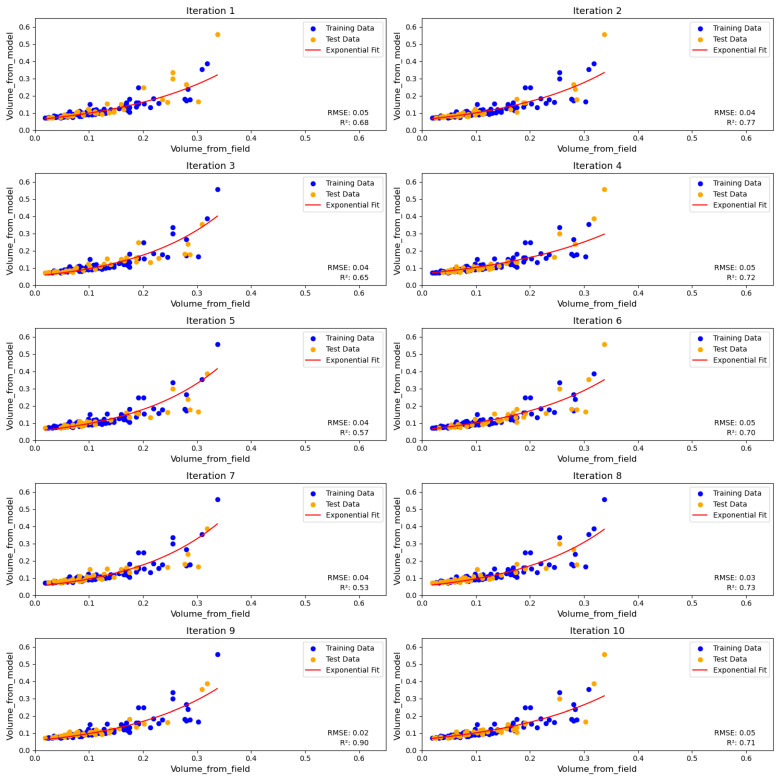
These plots show the accuracy assessment of the model by plotting the volumes computed by the model and the field measurements, respectively.

The empirical equations formulated were validated through two approaches. First, for 125 data points with recorded DBHs, we compared volumes computed from conventional allometric equations against those computed with our empirical equation. Employing a randomized cross-validation technique, we segregated the data into 70% training and 30% testing subsets, repeating this process 10 times (Figure 14). The resulting R^2^ values ranged from 045 to 0.84, showing moderate-to-strong predictive capability across data subsets.

**Figure 15:**
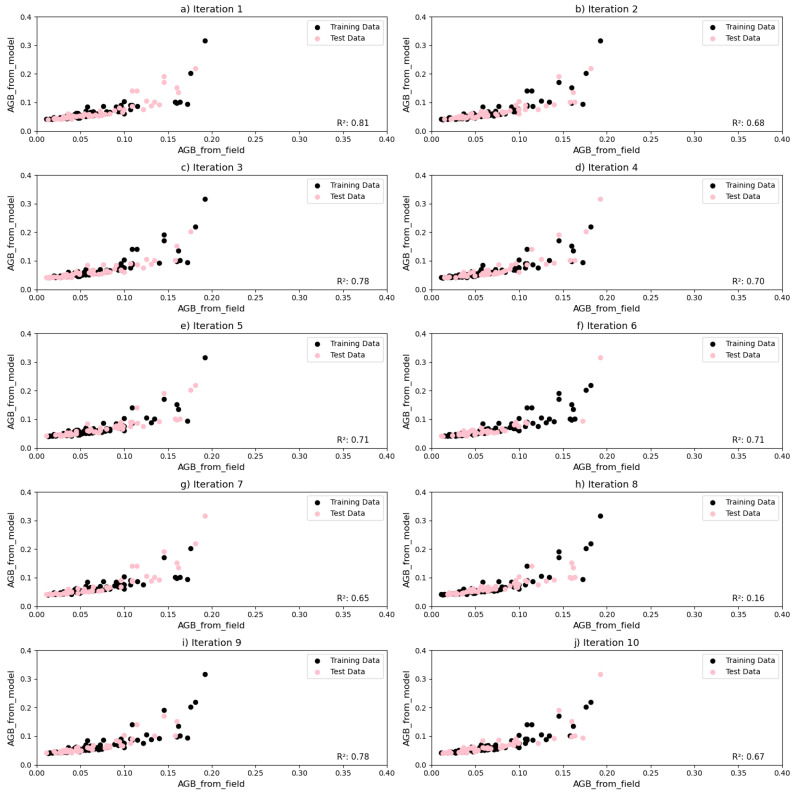
These plots show the accuracy assessment of the model by plotting the AGB computed by the model and the field measurements, respectively.

The AGB calculated from the stock volume computed by the model and the AGB measured from the field were also compared. Random iterations were carried out by dividing the dataset into 70:30 ratios for training and testing, respectively (Figure 15). The AGB recorded in the field varied from 0.02 to 0.2 tons per hectare, while the AGB computed from the model was between 0.1 and 0.4 tons/ha. The resulting R^2^ values ranged from 0.61 to 0.83, indicating moderate-to-strong predictive capability across different data subsets.

The developed model was tested for cumulative stock volume estimation per hectare in seven plots, each 25 m × 25 m in size, with 100 trees per plot. The tree crowns were delineated, respectively, for the seven plots.

After removing the overestimations and false positives of the tree crown canopies, the model estimated cumulative stock volumes between 190 and 208 cubic meters per hectare, while field measurements for 450 manually measured trees (out of 800 detected by the drone) ranged from 180 to 237 cubic meters per hectare. A strong correlation (R^2^ = 0.75) was found between the model and field data, with the relationship described by the Equation (2):

**y = 1.14x − 39.79,** (3)

where y represents field measurements, and x represents model predictions.

***Equation (3)**: Empirical equation developed using the stock volume and tree crown area.*

**Figure 16:**
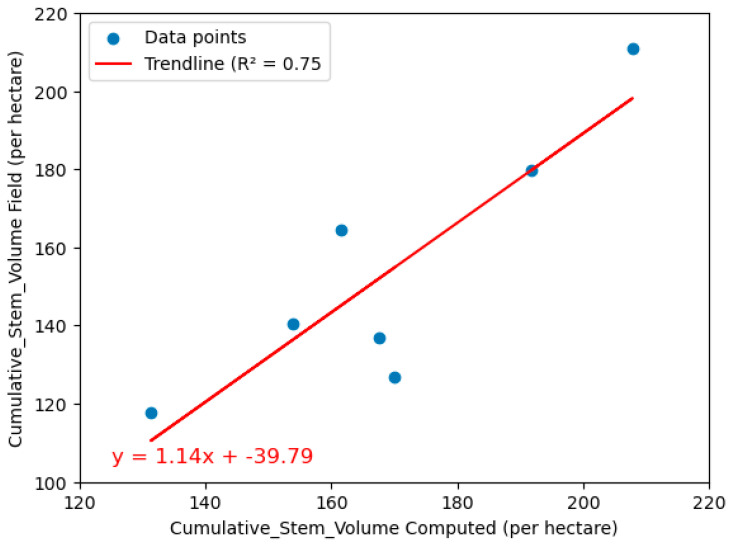
Validating the model for computing the cumulative stock volume.

These results demonstrate the model’s reliability for estimating tree and plot-level stock volumes, with potential applications in forest inventories (Figure 16).

The cumulative AGBs for the seven plots (Table 1) were also calculated and compared with the field, with measurements showing less difference between the values with an R^2^, which indicates the good functionality of the developed model.

*Challenges*

There were some challenges that were faced during the study. The drone imagery was not adequate to capture every small detail of the edges of the trees, which resulted in some misjudgments of the tree crowns. Also, the machine learning algorithm failed in exactly identifying some tree canopies due to dense canopy cover and canopy overlaps. At last, due to the uneven terrain, there were some errors in ground observation.

## 4. Discussions

The results of this study demonstrate the effectiveness of using Detectron2 for tree crown delineation and the subsequent computation of crown areas, which is a critical step in forest inventory assessments. The segmentation of tree crowns from drone imagery was accurate, with a total of 125 trees analyzed after excluding false positives and overlapping crowns. The positive correlation between the tree crown area and trunk circumference highlights the exponential growth of the crown area with increasing circumference. This relationship is particularly pronounced for circumferences beyond 0.25 m, where crown areas significantly expand. The calculation of the DBH from the trunk circumference for the estimation of stock volume using conventional allometric formula underscores the potential to automate the prediction of tree stock volume. The development of the model to predict the tree stem volume from the tree crown area yielded promising results. The positive correlation between crown area and stem volume shows that larger crown areas are associated with higher stem volumes. The application of a statistical measurement, such as the Z score, to filter the data and remove outliers improved the model’s accuracy. The optimal empirical equation derived from the training and testing sets demonstrated a good fit, with an R^2^ value of 0.6722. The estimation of AGB, ton carbon stock, and ton carbon dioxide emissions using the model showed close agreement with field measurements. The overall R^2^ of 0.41 with RMSEs of 0.04, 0.01, and 0.007, respectively, further confirms the model’s predictive capability. The validation of the empirical equations for stock volume computations through randomized cross-validation techniques yielded moderate-to-strong predictive capabilities across different data subsets, with R^2^ values ranging from 0.45 to 0.84. The comparison of AGB calculated from the model and the AGB measured from the field yielded R^2^ values ranging from 0.61 to 0.83, indicating the good predictive capability of the model. The application of the model for cumulative stock volume estimation per hectare in seven plots showed a strong correlation with field measurements, with an R^2^ value of 0.75. The equation derived from this comparison was y = 1.14x − 39.79. Also, the cumulative AGB for the seven plots, calculated from the model and compared with field measurements, showed minimal differences, with an R^2^ indicating the good functionality of the developed model. This consistency in AGB estimation at the plot level highlights the potential for scaling up the model’s application to larger forest areas.

## 5. Conclusions

The objective of this work was to perform tree crown segmentation for crown area estimation and determine the empirical relationship between tree crown area and stem stock volume, contributing to a better understanding of forest ecology and finally outlining a methodology for automatically predicting stock volume based on tree crown segmentation using UAV data. This work presents a non-invasive and efficient method for forest assessment that highlights the effectiveness of utilizing drone imagery and tree crown delineation techniques for estimating both individual tree volumes and cumulative stock volumes by developing an empirical model that automatically estimates stock volume using tree crowns. The model highlights a strong exponential correlation between crown area and stem stock volume, with a coefficient of determination of 0.67 and mean squared error (MSE) of 0.0015. It was found that the model represented here revealed a moderate-to-strong predictive performance, as evidenced by R^2^ values ranging from 0.45 to 0.84 for the volume measured on the field concerning the volume computed using empirical equations. Additionally, the model showed a significant correlation, with R^2^ = 0.75, for cumulative stock volume estimation using the tree crown area parameter.

These findings show that UAV-derived crown area measurements, combined with the empirical equation, offer a reliable and scalable method for stock volume computation and AGB estimation. Above-ground biomass estimations from field measurements compared to model predictions also have a lot less deviations with RMSE = 0.04.

AGB is crucial for understanding carbon storage and forest management, as it directly impacts carbon stock assessments and ecological modeling. By leveraging aerial remote sensing technology, this study provides an accurate, efficient approach for forest inventory, balancing precision with scalability in both stock volume and AGB estimation.

These findings suggest that UAV-derived crown area measurements, combined with the empirical equation, offer a reliable, scalable method for forest inventory, balancing accuracy with efficiency for stock volume computation by using aerial remote sensing technology.

## Figures and Tables

**Table 1:** The cumulative AGBs for the seven plots measured in tons/ha from field measurements and model predictions.

AGB Computed by the Model	AGB Computed from the Field
74.84	67.14
75.45	93.80
87.66	80.08
90.35	120.23
76.00	45.74
95.52	77.99
102.57	72.20

## Data Availability

Data are contained within the article.
